# Editorial: Affective, Cognitive and Social Neuroscience: New Knowledge in Normal Aging, Minor and Major Neurocognitive Disorders

**DOI:** 10.3389/fpsyg.2022.857475

**Published:** 2022-03-02

**Authors:** Rosalba Morese, Antonella Carassa, Sara Palermo

**Affiliations:** ^1^Faculty of Communication, Culture and Society, Università della Svizzera italiana, Lugano, Switzerland; ^2^Faculty of Biomedical Sciences, Università della Svizzera italiana, Lugano, Switzerland; ^3^Neuroradiology Unit, Diagnostic and Technology Department, Fondazione IRCCS Istituto Neurologico Carlo Besta, Milan, Italy; ^4^European Commission Innovation Partnership on Active and Healthy Ageing, Brussels, Belgium

**Keywords:** aging, cognitive decline, neurocognitive disorders, emotional recognition, anesthesia, dance, pain

## Introduction

The aging of the population is a phenomenon that is characterizing the demographic framework of any country, particularly those in the West and Japan. Population aging is a long-term trend that started several decades ago globally and especially in Europe with an increasing share of older people together with a decreasing share of people of working age in the global population (Eurostat, 2020). According to Eurostat, in 2080, the European population of the 28 member states will be just under 520 million people and almost 30% of them will be over 65 years old (about 10 percentage points more than the current situation). Increased life expectancy, improved economic conditions, technological and medical progress have changed the age structure of societies but not without consequences. The progressive aging population is forcing States to rethink the guarantees offered by the welfare system (de Meijer et al., 2013; Palermo, 2020). It is necessary to review the framework of social interventions in addition to the existing forms of economic assistance: health and home care and socialization services must be integrated with other new types of service, aimed at groups of citizens and patients who are particularly vulnerable (Bozzatello et al., 2019; Morese et al., 2019a,b; Morese and Longobardi, 2020; Palermo, 2021).

Furthermore, the first data on the COVID-19 emergency—collected at the beginning of the year 2020 in China and Italy—showed that the population group most severely tested for symptoms was the elderly. This was also confirmed in other countries, including the United States, as the epidemic spread to the rest of the world, becoming a pandemic. Starting with the USA data, and linking up with previous analyses, a study examined the positive/negative initiatives toward older people in the COVID-19 pandemic, and also the consequences these will have on the perception of aging in the immediate and in the long term (Monahan et al., 2020). The COVID-19 pandemic has highlighted the need to rethink three key areas: health care, employment policies and public awareness of aging. This is the only way to avoid discrimination against older people and to promote their active inclusion in society (Morese et al., 2019a; Corna et al., 2021; Palermo, 2021). In this context, it is becoming increasingly important to (a) know the changes that occur in the nervous system, cognitive and emotional-motivational functions in healthy and pathological aging; (b) know the epidemiological, clinical, and neuropsychological characteristics of dementia and other neurodegenerative pathologies (Palermo et al., 2018; Morese and Palermo, 2020); (c) be able to set up a thorough multidimensional assessment according to a bio-psycho-social approach; (d) be able to set up and manage prevention interventions and physical-cognitive enhancement in healthy and pathological aging; (e) promote socialization and social inclusion (Morese et al., 2016; Morese and Palermo, 2019).

To date, we know that does not exist a single direction of aging, but several kinds of ageotypes have been detected (Palermo, 2020). Physical and cognitive frailty and neurodegenerations have a major impact on higher-order cognitive functions and human organized behavior. Not surprisingly, therefore, the previous Research Topic received a favorable reception (Palermo et al., 2020a).

With this new call for Authors, we wanted to explore the continuum between normal aging and neurodegeneration. This Research Topic is part of a series of Research Topics on neuronal mechanisms and brain circuits that regulate the fundamental aspects of human behavior. Volume I: Perspective-taking, Self-awareness and Social Cognition in Neurodegenerative Disorders, cerebral abnormalities and Acquired Brain Injuries (ABI): A Neurocognitive Approach.

Our primary endpoint was to provide the reader with the most up-to-date perspective on how the interplay between physiological systems, neural mechanisms and neuropsychological processes lead to complex and highly organized behavior in normal aging and minor and major neurocognitive disorders. We aimed to provide an overview of new findings in aging by exploring the neuropsychological processes and brain circuits that regulate fundamental aspects of human behavior.

We have welcomed contributions from clinicians, neuroscientists, and academics. The goal we set for ourselves was to give an opportunity for researchers from different disciplines to discuss recent advances in this field. The authors have provided us with valuable suggestions for improving diagnostic and clinical practice. Their insights will be useful to imagine new research perspectives. The contributions here presented provide an interesting cross-section of the multidisciplinary hybridizations needed to study and manage aging.

## Aging: Normal Functioning and Cognitive Impairment

Aging first causes a change in motivation. Social relations play a central role in motivation in healthy aging. However, the study of emotion recognition and regulation in older people is a relatively new field. A study to investigate the cognitive functions of emotional facial recognition, and to assess the influences of depressive mood on emotion recognition in older participants, like to repeat the influences of aging on the emotional recognition was presented in this special issue (Ochi and Midorikawa). Age, emotional state, and cognitive function have predicted the ability to recognize and attribute valence to emotions. Moreover, paradoxical relationships between emotional face recognition and some verbal functions were also observed. A deficit in the recognition of emotional facial expressions could be an early indication of mild cognitive impairment. This conclusion is corroborated by the evidence that facial emotion recognition is indicative of alterations in metacognitive-executive functions linked to brain functional abnormalities across pathologies (for example, Palermo et al., 2015, 2017).

Social cognition impairment related to metacognitive-executive functions is often detectable in Alzheimer's Disease (AD). Original research found that it was associated with connectivity and with the prefrontal cortex (Valera-Bermejo et al.), alterations in the inter-network connection involving the fronto-parietal network, the salience network, the TPJ, and the insula appear to be present.

In the cognitive domain, it is well known that word-finding ability progressively declines in older people. The topic is addressed here by investigating the associations between word-finding ability and language-related components with cerebral aging status, investigated with the brain age paradigm (Chen et al.). Chronological and white matter aging seem to affect language differently, involving not only domain-specific processing but also executive functions.

Movement and walking also become increasingly difficult and uncertain with advancing age and are therefore increasingly indicated as early predictors of physical and cognitive frailty. Evidence of this is provided by original research on gait kinematic in older people with Subjective Cognitive Decline (SCD) and mild cognitive impairment (MCI) (Zhong Q. et al.). The authors, therefore, suggest conducting gait analysis in the clinical setting to identify MCI patients also at functional observation. This is a fundamental aspect: the personal motor repertoire—supported by the mirror neuron system (MNS)—is sensitive to neurodegeneration. Indeed, an interesting aspect has been indicated in the initial stages of Alzheimer's disease, for which motor and cognitive performance seem to be sustained by the hyperactivation of the central nervous system (Farina et al., 2020). Abnormalities in the MNS have also been identified in movement disorders in old age (Palermo et al., 2020b). More and more evidence suggests that movement improves executive functions in older people. Among these, metacognitive-executive functions are particularly important (Morese et al., 2018, 2020). Light-intensity aerobic dance (LADE) may be particularly suitable for the elderly population. In particular, intermittent LADE seems to be particularly appreciated by the elderly population and can contribute to the development of executive functions enhancement programs (Hyodo et al.). This line of research includes a meta-analysis aimed at studying the effectiveness of dance programs on cognition, psycho-behavioral symptoms and motor functions in MCI (Liu C. et al.). The authors found that dance interventions benefit cognition although the mechanisms by which this happens are still unknown. Previous research assumed that the modulation of the brain network activated in complex action observation and embodiment may be evaluated as an important finding for neuro-rehabilitative programs (Palermo et al., 2020b).

To date, the relationship between healthy lifestyle factors and autonomy in daily living is still unclear. This possible association was explored in original research with a view to primary prevention (Li D. et al.). Adherence to a healthy lifestyle was found to be linked with a lower risk of neurophysiological function limitation. Indeed, studies stress the value of creating and developing programs that can help to reverse the direction of frailty to break the vicious circle with cognitive impairment, especially executive dysfunction, and the progression to minor and major neurocognitive disorders (Bartoli et al., 2020a; Amanzio and Palermo, 2021; Palermo, 2021).

That sedentariness and bad habits affect cognition is supported by two further studies. In the first, it has been found that the relationships between cognitive decline, blood lipid levels, and obesity could be influenced by blood pressure and sex (Wang Y. et al.). In the second, it has been found that cigarette smoking causes functional connectivity disruption between the nucleus basalis of Meynert and precuneus in MCI patients. In turn, nicotine affects cognition through the cholinergic pathway (Qiu et al.).

## From Mild Cognitive Impairment to Major Neurocognitive Disorders

An important contribution to the understanding of the onset of neurocognitive disorders and their classification comes from the exploration of biomedical and laboratory variables. First and foremost is the evaluation of the CSF and genetics. In particular, Apolipoprotein E (APOE), glucose metabolism, lipids could take action with complex moderation interactions on cognitive function, probably acting on the same pathways involved in the pathogenesis of AD (Liu L. et al.).

A second original research (Hestad et al.) investigated whether the effects of APOE e4 allele can lead to increased brain functional vulnerability and brain reserves reduction, in this way mediating the progress from the aging brain to major neurocognitive disorders.

In the second original research, Authors (Zhong J. et al.) found some specific protein markers that could be useful to early evaluate cognitive decline, also early a clinical diagnosis of AD.

A preclinical important subtle symptom has come back into the limelight because of the pandemic: anosmia. Indeed, odor identification is common in neuropsychiatric symptoms associated with progression from MCI to AD. Original research found that patients with behavioral changes showed significantly worse performance in odor identification and cognition, with cognitive alterations which in turn worsen the ability to discriminate by smell (Wang Q. et al.).

What trajectory the neurodegenerative processes take will also depend in part on the individual's cognitive reserve (Gallo et al.). Brain integrity evaluation combined with residual cognitive performance represents a fruitful approach to cognitive reserve evaluation.

## Vascular and Ischemic Diseases

Long-term systolic blood pressure variability and mean heart rate are independent predictors of cognitive impairment and its worsening in patients at high cardiovascular risk. Since heart rate fragmentation (HRF) is linked with increasing age and cardiovascular problems, it becomes essential to investigate whether disrupted cardiac neuroautonomic function would be correlated with poor cognitive function and more severe cognitive decline (Costa et al.). The Authors found that amplified HRF in sleep was linked with reduced cognitive performance and with more cognitive deterioration. Sleep is also analyzed as the main predictor in a study exploring the bidirectional relationship between sleep duration and cognitive function (Hua et al.).

Dysfunctions after stroke are linked to white matter microstructure damage (Wei et al.). The authors found that pontine infarction results in widespread white matter tracts disruption which is associated with impaired motor and cognitive functions in the infarcted.

Considering disease progression, vascular dementia is the second most common cause of major neurocognitive disorder in the elderly. For this reason, it has received particular attention from researchers over the years. The study of the effects of vascular brain damage sees its initial expression in single case studies that allow a first approach to the functional and neuropsychological modeling of damage. Various examples can be found in the literature (for example, Palermo et al., 2014; Bartoli et al., 2020b).

We have ventured into exploring the effects of a blood disruption to the brain in this Research Topic as well. In addition to the study by Hestad et al., other research groups have contributed to the topic. Indeed, combing neuroimaging findings and neuropsychological aspects can integrate complementary evidence for the early detection of MCI (Li X. et al.). This possibility has been explored thanks to a propensity score matching approach. The authors found that cerebral small vessel disease (CSVD) can be considered an independent risk factor of MCI.

The study of the effects of vascular brain damage sees its initial expression in single case studies that allow a first approach to the functional and neuropsychological modeling of damage. Various examples can be found in the literature.

Subcortical ischemic vascular disease (SIVD) represents an important problem for large cases of patients hospitalized for cerebrovascular disease and underlies dysfunctions in executive functions. Distinct mechanisms of memory impairment differentiated AD from SIVD, particularly prospective memory. For the first time, a study based on a neurocognitive approach ascertained a frontal dysconnectivity associated with a deficit in prospective memory in SIVD (Zhuang et al.).

## Anesthesia and Post-Operative Neurocognition

It differs in approach from previous articles, a narrative review of the effects of the sevoflurane, a common anesthetic for surgical patients (Wang C. et al.). Treatment with sevoflurane can increase cases of postoperative cognitive dysfunction (POCD), while POCD patients show cognitive dysfunctions after surgery. Better understanding the role of mechanism of sevoflurane-induced POCD could help programs for prevention and treatment, especially in the elderly. With this aim, the Authors reviewed the diagnosis of POCD, and the possible mechanisms of sevoflurane-induced POCD, introduce animal models into clinical research, summarize treatment progresses.

On this thread, a clinical trial on the role of anesthesia management for the post-operative cognitive function in older surgical patients (Yang et al.) show interesting results; they seem promising in that the proposed approach reduces perioperative neurocognitive disorder while improves functional connectivity by reducing systemic inflammation.

## Pain and Cognitive Impairment

Pain is the most frequent reason for patients to seek medical attention. Chronic pain conditions have been associated with cognitive impairment, affecting attention, working and long-term memory, information processing, and reasoning (Moriarty et al., 2011). For the first time, cognitive impairment has been assessed in patients with burning mouth syndrome (BMS) (Canfora et al.). BMS was here associated with working memory, attention, and also executive dysfunctions.

## Author Contributions

All authors listed have made a substantial, direct, and intellectual contribution to the work and approved it for publication.

## Conflict of Interest

The authors declare that the research was conducted in the absence of any commercial or financial relationships that could be construed as a potential conflict of interest.

## Publisher's Note

All claims expressed in this article are solely those of the authors and do not necessarily represent those of their affiliated organizations, or those of the publisher, the editors and the reviewers. Any product that may be evaluated in this article, or claim that may be made by its manufacturer, is not guaranteed or endorsed by the publisher.
